# Vimentin and Ki67 expression in circulating tumour cells derived from castrate-resistant prostate cancer

**DOI:** 10.1186/s12885-016-2192-6

**Published:** 2016-02-29

**Authors:** C. R. Lindsay, S. Le Moulec, F. Billiot, Y. Loriot, M. Ngo-Camus, P. Vielh, K. Fizazi, C. Massard, F. Farace

**Affiliations:** INSERM U981, University of Paris-Sud XI, Translational Research Laboratory, Gustave Roussy Cancer Campus, Villejuif, France; Department of Medical Oncology, Bergonie Cancer Institute, Bordeaux, France; Department of Medical Oncology, Gustave Roussy Cancer Campus, Villejuif, France; Department of Biopathology, Gustave Roussy Cancer Campus, Villejuif, France

**Keywords:** Prostate, Vimentin, Ki67, Circulating

## Abstract

**Background:**

High circulating tumor cell (CTC) counts are associated with poor prognosis in advanced prostate cancer, and recently CTC number was suggested to be a surrogate for survival in metastatic castrate-resistant prostate cancer (mCRPC). Ki67 and vimentin are well-characterised markers of tumour cell proliferation and the epithelial-mesenchymal transition (EMT), respectively. Here we asked if the expression of vimentin and Ki67 in CTCs offered prognostic or predictive information in mCRPC.

**Methods:**

In two separate patient cohorts, anti-vimentin or anti-Ki67 antibodies were added to the free channel in the CellSearch® system for analysis of peripheral blood samples. For each cohort, association of CTC number with clinical characteristics were assessed using Fisher’s exact, Mann-Whitney and chi-squared tests. Kaplan-Meier method and log-rank tests were used to analyse overall survival (OS) of vimentin-expressing and Ki67-expressing CTC patient cohorts.

**Results:**

In this retrospective analysis, CTC vimentin expression was analysed in 142 blood samples from 93 patients, and CTC Ki67 expression was analysed in 90 blood samples from 51 patients. In the vimentin cohort, 80/93 (86 %) of baseline samples from patients were CTC-positive overall (≥1 total CTC per 7.5 mls blood), and 30/93 (32.3 %) vimentin CTC-positive (≥1 vimentin-positive CTC per 7.5 mls blood). 41/51 (80.4 %) of baseline samples from patients in the Ki67 cohort were CTC-positive overall, and 23/51 (45.1 %) Ki67 CTC-positive (≥1 Ki67-positive CTC per 7.5 mls blood). There was no significant difference in baseline PSA in patients with vimentin-positive CTC at baseline versus those with no vimentin-positive CTC at baseline (*p* = 0.33). A significant reduction in OS was shown in patients with vimentin-positive CTC compared to those without vimentin-positive CTC (median 305 days vs 453 days, *p* = 0.0293). There was no significant difference in baseline PSA in patients with Ki67-positive CTC at baseline versus those without Ki67-positive CTC (*p* = 0.228), but OS was significantly reduced in the Ki67-positive CTC group (median 512 days vs 751 days, *p* = 0.0091). No changes in relative proportion of vimentin- or Ki67-positive CTCs were observed in post-treatment samples compared to baseline.

**Conclusions:**

Analysis of vimentin and Ki67 expression can straightforwardly be assessed in CTCs from patients with mCRPC. Poorer survival outcomes were observed in vimentin- and Ki67-positive CTC patients.

**Translational study protocols:**

CEC-CTC (IDRCB2008-AOO585-50) and Petrus (NCT01786031).

**Electronic supplementary material:**

The online version of this article (doi:10.1186/s12885-016-2192-6) contains supplementary material, which is available to authorized users.

## Background

Circulating tumor cells (CTCs), captured as a ‘liquid biopsy’ from blood for enumeration and biological characterization of cancers, have the potential to replace biopsy and provide important clinical information on prognosis, therapeutic choice, and drug resistance, while also being of interest for drug development and biomarker discovery. They may represent an alternative source of tumor tissue which is easily accessible using a simple blood test, allowing longitudinal monitoring of tumor aggression and biology at different timepoints to guide therapeutic decisions in a patient’s treatment course [1–3]. Prostate cancer was one of the first malignancies where the prognostic value of monitoring CTC numbers was demonstrated in patients with advanced disease, both before and during treatment for castrate-resistant prostate cancer (CRPC) using the FDA-approved CellSearch technology [4, 5]. Moreover, a recent prospective trial demonstrated that CTC count and LDH value could be a surrogate of overall survival in a population of mCRPC patients treated with abiraterone in the COU-301 trial [6]. The potential of CTC for molecular characterization has been demonstrated on a number of occasions, most recently in CRPC with the demonstration of their variable androgen receptor (AR) expression [7–12].

Ki-67 is a nuclear protein that is associated with ribosomal RNA synthesis and may be necessary for cell cycle proliferation. Its tissue staining has consistently demonstrated prognostic value in prostate cancer, and has been tested in men managed with radiation and surgery, as well as in those conservatively managed without definitive therapy [13–16]. One group of studies used pretreatment biopsies of patients undergoing radiation and androgen deprivation as part of the Radiation Therapy Oncology Group (RTOG) 92–02 trial, defining a cut-off of 11.3 % high Ki-67 staining as independently correlated with an increase risk in distant metastasis, cancer-specific death, and overall death [13, 14]. A Mayo clinic study described similar outcomes from patient tissue taken before or during definitive prostatectomy, using a staining threshold of 6 % high Ki67 (present in 11 % of patients) which correlated with increased risks of cancer progression and cancer-specific mortality [16]. The phase III GETUG 12 study, which assessed androgen-deprivation therapy in high risk localized prostate cancer, showed that high Ki67 predicted an unfavourable PFS using a median Ki67 cutoff of 1 % [17]. Finally, 243 conservatively-treated patients from the Transatlantic Prostate Group were shown to have significantly increased risk of cancer-specific mortality in the 5 % of patients who had >10 % Ki67 staining [15]. The detection of Ki67 in CTCs has been previously reported using a microfluidic CTC detection method, offering the opportunity to assess if longitudinal quantification of proliferative and non-proliferative subpopulations may be more clinically informative than examination of total CTC count and/or biopsy alone [18].

Vimentin is a filamental protein expressed in mesenchymal cells, often recorded as a marker of tumour cell invasion via its expression during activation of the epithelial-mesenchymal transition (EMT) [19, 20]. Compared to Ki67, its use in biological interrogation of CTC is more commonplace. Interest in EMT/vimentin in the CTC field continues due to the continuing biological challenge it represents: CTC undergo EMT in order to enter the bloodstream where they are captured, yet many current enrichment methods and definitions of CTC do not incorporate mesenchymal markers such as vimentin. It is hypothesized that, in cancers such as non-small cell lung cancer which have a low yield of CTC via EPCAM (epithelial)-antibody enrichment in CellSearch, many ‘EMT’ and mesenchymal CTC are missed as a consequence of sub-optimal enrichment methods [21–24]. Despite this potential problem, the validity of EMT analysis using the CellSearch platform has previously been confirmed by a report documenting the co-expression of vimentin with cytokeratin in samples from 10/10 CRPC patients, with 108/126 CTCs in total co-expressing these markers [25].

Given the association of Ki67 and vimentin with poor prognosis in metastatic CRPC biopsy, we hypothesized that vimentin or Ki67 expression in CTC would offer a poorer prognostic picture compared to that observed in patients with no vimentin- or Ki67-positive CTCs. We analysed Ki67 and vimentin expression in CTC derived from a total of 144 advanced prostate cancer patients using CellSearch technology, optimized and validated to assay Ki67 and vimentin in separate patient cohorts. Our results corroborate those seen in tumour biopsies, with a diminished OS observed in patients with vimentin-expressing CTCs and those with Ki67-expressing CTCs.

## Methods

### Patients

Peripheral blood (7.5 ml) was collected from metastatic CRPC patients recruited to one of two Gustave-Roussy CTC translational study protocols, CEC-CTC (IDRCB2008-AOO585-50) and Petrus (NCT01786031). For eligibility, patients had to have a confirmed diagnosis of metastatic CRPC (either by contemporaneous or historical biopsy). All patients offered their written informed consent and were enrolled on institutional protocols approved by the local Gustave-Roussy ethics review committee. The following minimal clinical data were collected: CTC count at baseline and following their first cycle of treatment (at cycles 2–3), date of CTC sample, date of progression or death (if applicable), histology, metastatic sites, systemic treatment due at time of baseline CTC sampling, and previous treatment. Routine laboratory analyses were also performed on patients at baseline and at first blood sample after initiation of treatment, including prostate-specific antigen (PSA) for response assessment. Patients were recruited for the analysis between December 2010 until November 2014.

### CellSearch

Collection of blood and immunofluorescent (IF) staining of CTC was performed using the CellSearch® system (Janssen Diagnostics, LLC), as previously reported [4, 26]. Candidate cells (CTC +/− vimentin, or CTC +/− Ki67 – all cytokeratin positive) were identified using the CellTracks Analyzer II (Janssen Diagnostics, LLC) according to methods and IF expression criteria previously reported [27]. The number of CTCs is presented per 7.5 ml of blood. Unless otherwise stated in the text, patients in this study were defined as Ki67- or vimentin-CTC positive if they had ≥1 vimentin-positive or Ki67-positive CTCs. For Additional file 1: Figure S3, two separate patient cohorts were assessed in exploratory analyses: for (a) and (c), patients with ≥5 total CTCs and ≥1 vimentin-positive or Ki67-positive CTC were compared with those patients with ≥5 total CTCs and no vimentin-positive or Ki67-positive CTC; for (b) and (d), the 10 CTC-positive patients with the highest proportions of vimentin/Ki67-positive CTCs were compared with the 10 CTC-positive patients with the lowest proportions of vimentin/Ki67-positive CTCs.

### Ki67 and vimentin validation

This was established using the CellSearch platform: in separate experiments, FITC- labelled anti-vimentin antibody (Santa-Cruz, ’vimentin cohort’) and anti-Ki67 antibody (BD Biosciences, ’Ki67 cohort’) were added to the free channel in the CellSearch system. Anti-vimentin was tested using donor blood spiked with A549 (vimentin + ve) and T47D (vimentin -ve) cancer cell lines (Additional file 1: Figure S1a). Anti-Ki67 was tested in donor blood spiked with A549 and SKBR3 cancer cell lines: in these cell lines, phytohaemagglutinin A was used to stimulate proliferation and anti-IgG was used as a negative antibody control (Additional file 1: Figure S1b). CellSearch runs were carried out at three different exposure (0.4 s, 0.6 s, 0.8 s) with no difference evident, thus 0.8 s was chosen as standard for analysis (data not shown). Experiments were repeated in duplicate.

### Statistics

The association of CTC number with clinical characteristics and patient demographics were assessed using Fisher’s exact test for dichotomous factors, and Mann-Whitney tests for continuous data. The Kaplan-Meier method was used to estimate overall survival, and the difference between CTC cohorts compared using the log-rank test. Chi-square tests were used to analyse categorical data. Data cutoff for survival estimates was March 1st, 2015: outcomes were censored if a patient had not died before this date. Overall survival is defined as the time from first CTC sample until death from any cause, cancer-related or otherwise. Statistics were analysed using GraphPad Prism 6.03 for Windows. All p values were two sided and considered statistically significant at <0.05.

## Results

### Patient characteristics

**Fig. 1:**
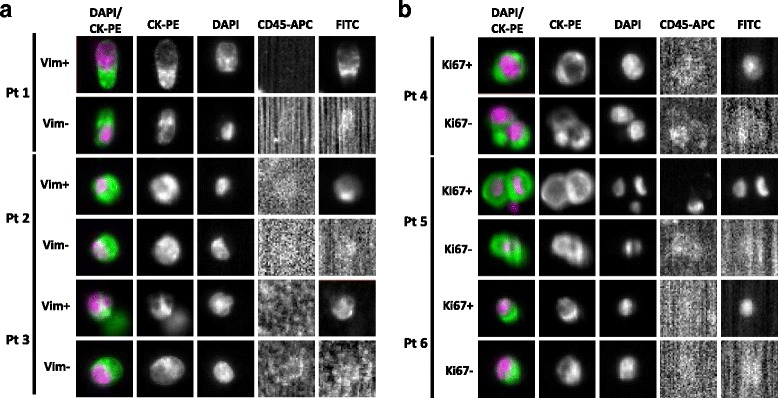
Vimentin- and Ki67-expressing CTCs from castrate-resistant prostate cancer patients Representative pictures from CellSearch system of vimentin staining (**a**) and Ki67 staining (**b**) in CTCs from three patients in each analysis. Pt=patient, vim=vimentin

As only one free channel position for either Ki67 or vimentin antibodies was available using CellSearch, analyses were conducted in separate patient cohorts: a ‘vimentin cohort’ and a ‘Ki67 cohort’. Patient details from each are recorded in Tables 1 and 2. From the vimentin cohort, 93 patients in total were recruited: 63 patients were vimentin CTC-negative (no vimentin-positive CTCs) at baseline and 30 patients were vimentin CTC-positive (≥1 vimentin-positive CTC) (Tables 1 and 3, Fig. 1a). Both vimentin patient cohorts had similar baseline characteristics, other than an increase in visceral/multiple metastatic sites for patients in the vimentin CTC-positive group (43.3 vs 30.2 %) which was consistent with more patients in this group reported to be receiving at least a 3rd line of systemic treatment (40 vs 22.2 %) (Table 1). The majority across both groups were post-docetaxel and due to receive at least 2^nd^ line systemic treatment (Table 1).

**Table 1:** Baseline characteristics of patients with prostate cancer according to Vimentin CTC status

Characteristic	Vimentin CTC-negative patients (no vimentin + ve CTCs) (%)	Vimentin CTC-positive patients (≥1 vimentin + ve CTC) (%)
Total patient numbers	63	30
Age, years		
Median	76	72
Range	48–93	53–86
Number of metastasic sites		
1	26/63 (41.3)	11/30 (36.7)
2	21/63 (33.3)	9/30 (30)
3+	15/63 (23.8)	10/30 (33.3)
Not stated	1/63 (1.6)	0/30 (0)
Metastasic sites		
Lymph Nodes alone	3/63 (4.8)	1/30 (3.3)
Bone alone	24/63 (38.1)	10/30 (33.3)
Bone and lymph nodes	17/63 (27)	6/30 (20)
Visceral/multiple sites	19/63 (30.2)	13/30 (43.3)
Treatment at time of 1^st^ CTC specimen		
Cabazitaxel	7/63 (11.1)	7/30 (23.3)
Docetaxel	7/63 (11.1)	3/30 (10)
Abiraterone	26/63 (41.3)	11/30 (36.7)
Enzalutamide	5/63 (7.9)	2/30 (6.7)
Radium 223	2/63 (3.2)	2/30 (6.7)
Others/Not Stated	16/63 (25.4)	5/30 (16.7)
Line of treatment at time of 1^st^ CTC specimen		
1	10/63 (15.9)	4/30 (13.3)
2	17/63 (27)	8/30 (26.7)
3+	14/63 (22.2)	12/30 (40)
Not stated	22/63 (34.9)	6/30 (20)
Previous treatments		
Primary surgery	10/63 (15.9)	7/30 (23.3)
Primary radiotherapy	27/63 (42.9)	12/30 (40)
Docetaxel	42/63 (66.7)	23/30 (76.7)
Enzalutamide	2/63 (3.2)	4/30 (13.3)
Cabazitaxel	10/63 (15.9)	7/30 (23.3)
Abiraterone	21/63 (33.3)	12/30 (40)
Total CTC levels at baseline		
Mean	98.8	492.6
Range	0–1185	1–4310

**Table 2:** Baseline characteristics of patients with prostate cancer according to Ki67 CTC status

Characteristic	Ki67 CTC-negative patients (no Ki67 + ve CTCs) (%)	Ki67 CTC-positive patients (≥1 Ki67 + ve CTC) (%)
Total patient numbers	28	23
Age, years		
Median	79.5	72
Range	63–90	52–89
Number of metastasic sites		
1	12/28 (42.9)	8/23 (34.8)
2	10/28 (35.7)	8/23 (34.8)
3+	4/28 (14.3)	7/23 (30.4)
Not stated	2/28 (7.1)	0/23 (0)
Metastasic sites		
Lymph Nodes alone	0/28 (0)	0/23 (0)
Bone alone	12/28 (42.9)	8/23 (34.8)
Bone and lymph nodes	8/28 (28.6)	5/23 (21.7)
Visceral/multiple sites	6/28 (21.4)	10/23 (43.5)
Unknown/Not stated	2/28 (7.1)	0/23 (0)
Treatment at time of 1^st^ CTC specimen		
Cabazitaxel	0/28 (0)	6/23 (23.1)
Docetaxel	6/28 (21.4)	3/23 (13)
Abiraterone	13/28 (46.4)	11/23 (47.8)
Others/Not stated	9/28 (32.1)	3/23 (13)
Line of treatment at time of 1^st^ CTC specimen		
1	5/28 (17.9)	3/23 (13)
2	8/28 (28.6)	5/23 (21.7)
3+	3/28 (10.7)	7/23 (30.4)
Not stated	12/28 (42.9)	8/23 (34.8)
Previous treatments		
Primary surgery	5/28 (17.9)	3/23 (13)
Primary radiotherapy	6/28 (21.4)	5/23 (21.7)
Docetaxel	21/28 (75)	20/23 (87)
Enzalutamide	1/28 (3.6)	0/23 (0)
Cabazitaxel	1/28 (3.6)	0/23 (0)
Abiraterone	2/28 (7.1)	5/23 (21.7)
Total CTC levels at baseline		
Mean	12.9	92.8
Range	0–79	2–316

**Table 3:** Baseline CTC characteristics of patients with prostate cancer according to vimentin and Ki67 CTC status

Vimentin cohort	
Number of pts with ≥1 CTC (%)	
Total CTC	80/93 (86)
Vimentin CTC	30/93 (32.3)
Number of pts with ≥5 CTC (%)	
Total CTC	69/93 (74.2)
Vimentin CTC	10/93 (10.8)
Number of pts with ≥5 CTC and ≥1 vimentin CTC	28/69 (40.6)
Number of pts with only Vimentin-positive CTC	1/93 (1.1)
Number of Vimentin-positive CTCs in total	960/26299 (3.7)
Ki67 cohort	
Number of pts with ≥1 CTC (%)	
Total CTC	41/51 (80.4)
Ki67 CTC	23/51 (45.1)
Number of pts with ≥5 CTC (%)	
Total CTC	35/51 (68.6)
Ki67 CTC	12/51 (23.5)
Number of pts with ≥5 CTC and ≥1 Ki67 CTC	22/35 (62.9)
Number of pts with only Ki67-positive CTC	3/51 (5.9)
Number of Ki67-positive CTCs in total	307/2495 (12.3)

From the Ki67 cohort, 51 patients in total were recruited: 28 patients were Ki67 CTC-negative (no Ki67-positive CTCs) and 23 patients were Ki67 CTC-positive at baseline (≥1 Ki67-positive CTCs) (Tables 2 and 3, Fig. 1b). The majority of patients across the cohort were beyond 1st line systemic treatment. For the Ki67 CTC-positive group, increases in patients with ≥3 metastatic sites (30.4 vs 14.3 %) and visceral metastatic sites (43.5 vs 21.4 %) were again consistent with more patients in this group reported to be receiving at least a 3rd line of systemic treatment (30.4 vs 10.7 %) (Table 2).

### CTC characteristics

Table 3 provides a summary of CTC characteristics at baseline within the 2 cohorts. Vimentin expression was analysed in 142 samples from 93 patients: all patients were sampled at baseline, 49 of whom were also sampled at later timepoints during the same treatment regimen. 80/93 (86 %) of baseline samples from patients were CTC-positive overall, and 30/93 (32.3 %) vimentin CTC-positive. 69/93 (74.2 %) patients belonged to the previously described ‘unfavourable’ prognostic category (≥5 standard CTCs) for overall CTC count, while 10/93 (10.8 %) patients had samples with ≥5 vimentin-positive CTCs [4]. In the unfavourable prognostic category, vimentin-positive CTC prevalence was higher (28/69 samples, 40.6 %). Only 1/93 patients (1.1 %) had samples containing vimentin-positive CTCs exclusively, with the other 29/30 vimentin-positive CTC samples harbouring at least one vimentin-negative CTC. 960/26299 (3.7 %) of CTCs were vimentin-positive across baseline samples from the cohort.

For Ki67 expression, 90 samples from 51 patients were analysed: all patients were sampled at baseline, 39 of whom were also sampled at later timepoints during the same treatment regimen. 41/51 (80.4 %) of baseline samples from patients were CTC-positive overall, and 23/51 (45.1 %) Ki67 CTC-positive. 35/51 (68.6 %) patients belonged to the ‘unfavourable’ prognostic category (≥5 standard CTCs) for overall CTC count, while 12/51 (23.5 %) patients had samples with ≥5 Ki67-positive CTCs. In the unfavourable prognostic category, Ki67-positive CTC prevalence was higher (22/35 samples, 62.9 %). 3/51 patients (5.9 %) had samples containing Ki67-positive CTCs exclusively, with the other 20/23 Ki67-positive CTC samples harbouring at least one Ki67-negative CTC. 307/2495 (12.3 %) of CTCs were Ki67-positive across first samples from the cohort.

### Prognostic impact of vimentin/Ki67 CTC expression

We first sought to ensure that, in line with that previously described, a prognostic difference was evident across both cohorts between those patients in the unfavourable CTC-positive prognostic category (≥5 standard CTCs per 7.5 ml) and those in the favourable CTC-positive prognostic category (<5 standard CTCs per 7.5 ml) at baseline sampling.

Across the vimentin and Ki67 cohort, baseline PSA was significantly higher in those patients who had unfavourable prognosis at baseline compared to those who were in the favourable category (mean PSA 671.2 vs 278.3, *p* = <0.0001) (Additional file 1: Figure S2a). A significant difference in median OS was also evident in patients who were in the unfavourable category at baseline compared to those in the favourable group (375 vs 712 days, respectively; HR 2.15, 95 % CI 1.35–3.04, *p* = 0.001) (Additional file 1: Figure S2b). We thus validated that the prognostic value of standard CTCs in our two cohorts was consistent with what has been reported previously [4, 5].

**Fig. 2:**
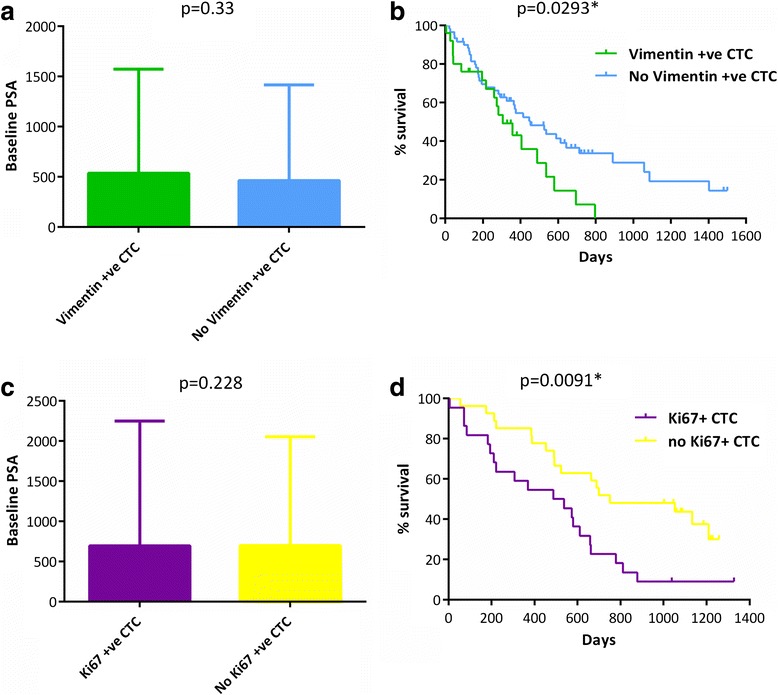
Prognostic impact of vimentin- and Ki67-expressing CTC. **a**-**b** Prognostic comparison of patients with vimentin-expressing CTC at baseline compared to those with no vimentin-expressing CTC, as assessed by bar charts comparing baseline PSA (**a**), and Kaplan-Meier plots of overall survival (**b**). **c**-**d** Prognostic comparison of patients with Ki67-expressing CTC at baseline compared to those with no Ki67- expressing CTC, as assessed by bar charts comparing baseline PSA (**c**), and Kaplan-Meier plots of overall survival (**d**). Mann-Whitney tests performed for (**a**) and (**c**), log-rank tests for (**b**) and (**d**); Bar charts show mean +/- standard deviation, *p*=<0.05

We next asked whether the baseline presence of vimentin- or Ki67-expression in CTC could have prognostic implications. Baseline PSA was not significantly different in those patients who were CTC-vimentin positive at baseline compared to those who had no vimentin-positive CTCs (mean PSA 537.0 vs 463.2, respectively; *p* = 0.33) (Fig. 2a). However a statistically significant difference in OS was evident between patients with vimentin-positive CTC and those with no vimentin-positive CTC (median 305 days vs 453 days, respectively; HR 1.8, 95 % CI 1.09–3.83, *p* = 0.0293) (Fig. 2b). When sub-divided into patients belonging to the unfavourable prognostic category of ≥5 standard CTCs overall, the presence of 1 vimentin-positive CTC did not diminish survival further (median 283 days vs 283 days, HR 1.33, 95 % CI 0.72–2.57, *p* = 0.347), and there was also no survival difference evident when the patients with the highest proportion of vimentin-positive CTCs were compared to those with the highest proportion of vimentin-negative CTCs (median 358 days vs 174 days, respectively; HR 1.36, 95 % CI 0.47–4.13, *p* = 0.569) (Additional file 1: Figure S3a, b).

In the Ki67 cohort, baseline PSA was not significantly different in those patients who were CTC Ki67-positive at baseline compared to those who had no CTC Ki67-positive CTC (mean PSA 695.7 vs 697.6, respectively; *p* = 0.228) (Fig. 2c). A statistically significant difference in OS was evident between patients with Ki67-positive CTC and those with no Ki67-positive CTC (median 512 days vs 751 days, respectively; HR 2.37, 95 % CI 1.35–5.23, *p* = 0.0091), although it was not clear that the presence of 1 Ki67-positive CTC diminished survival further when patients were sub-divided into the unfavourable prognostic category of ≥5 standard CTCs overall (median 537 days vs 725 days, HR 2.02, 95 % CI 0.89–4.24, *p* = 0.099) (Fig. 2d and Additional file 1: Figure S3c). There was also no survival difference evident when the patients with the highest proportion of Ki67-positive CTCs were compared to those with the highest proportion of Ki67-negative CTCs (median 600 days vs 664 days, respectively; HR 1.29, 95 % CI 0.52–3.33, *p* = 0.581) (Additional file 1: Figure S3d). Overall, this suggested that the presence of vimentin-positive or Ki67-positive CTC is more clinically important than their relative numbers or proportions, and that their presence in the early stages of advanced disease could be of greater prognostic significance than in later stages.

### Predictive impact of vimentin/Ki67 CTC expression

As vimentin expression has been associated with stem cell properties and treatment resistance in CTCs, we hypothesized that the mesenchymal profile of CTCs may become more apparent during treatment, even if overall CTC numbers were to diminish. We also questioned if the proliferative capacity of CTC, as represented by Ki67 expression, would be diminished by treatment, particularly those treatments with anti-proliferative properties such as docetaxel and cabazitaxel.

**Fig. 3:**
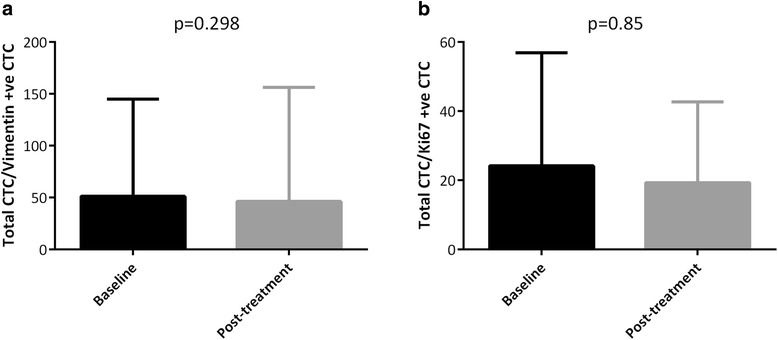
Change in vimentin- and Ki67-expressing CTC after initiation of treatment **a**) Bar chart assessing proportion of vimentin-positive CTC expression, compared to total CTC expression, at baseline and after treatment start **b**) Bar chart assessing proportion of Ki67-positive CTC expression, compared to total CTC expression, at baseline and after treatment start. ‘Post-treatment’ samples were taken at cycles 2-3 following initiation of treatment. Mann-Whitney test; Bar charts show mean +/- standard deviation, *p*=<0.05

We first tested whether the proportion of patients with vimentin-expressing CTC had altered between baseline CTC samples and paired follow-up CTC samples taken at cycles 2–3. When using ≥1 vimentin-positive CTC as a cutoff, there was no difference in patient proportion observed: 30/93 patients harboured vimentin-expressing CTC before treatment, compared to 19/49 patients after treatment (32.3 vs 38.8 %, *p* = 0.437) (Table 4). There was also no difference evident using ≥5 vimentin-positive CTC as a cutoff (10/93 patients vs 7/49 patients, 10.8 vs 14.3 %, *p* = 0.558) (Table 4). We next asked if the proportion of vimentin-expressing CTC may change with treatment in vimentin-positive CTC patients: ratio of total CTC/vimentin-positive CTC was not significantly different before and after treatment (mean 51 at baseline vs 46.2 after treatment; *p* = 0.298) (Fig. 3a).

**Table 4:** Change in patient Ki67 and vimentin CTC profile with treatment. Chi-squared test to analyse CTC frequencies in treatment groups. Samples from the second column were taken at cycles 2–3 following initiation of treatment

	Baseline sample before treatment (%)	First sample after treatment start (%)	
Number of patients with Ki67 + ve CTC (%)	23/51 (45.1)	15/39 (38.5)	*p* = 0.528
Number of patients with ≥5 Ki67 + ve CTC (%)	12/51 (23.5)	6/39 (15.4)	*p* = 0.336
Number of patients with Vim + ve CTC (%)	30/93 (32.3)	19/49 (38.8)	*p* = 0.437
Number of patients with ≥5 Vim + ve CTC (%)	10/93 (10.8)	7/49 (14.3)	*p* = 0.558

For Ki67 CTC testing, no difference in patient proportion harbouring Ki67-positive CTC was observed before and after treatment, regardless of whether ≥1 Ki67-positive CTC or ≥5 Ki67-positive CTC was used as a cutoff (≥1 CTC: 23/51 patients before vs 15/39 patients after, 45.1 vs 38.5 %, *p* = 0.528; ≥5 CTC: 12/51 patients before vs 6/39 patients after, 23.5 vs 15.4 %, *p* = 0.336) (Table 4). The proportion of Ki67-expressing CTCs relative to Ki67-negative CTC did not significantly change (mean 24.1 at baseline vs 19.3 after treatment; *p* = 0.85) (Fig. 3b). Thus, overall there was no significant change in proportions of Ki67/vimentin patients or CTCs between baseline and post-treatment samples.

## Discussion

Here we report CTC expression of vimentin and Ki67 in advanced/metastatic prostate cancer, offering a clinical insight into their mesenchymal and proliferative properties. We have established a method for testing CTC expression of Ki67 and vimentin in the normal pathway of prostate cancer care. In line with previous studies, we confirmed the prognostic value of detection of ≥5 standard CTCs at baseline through analysis of PSA and OS. No significant difference was evident when baseline PSA was split into vimentin-positive and Ki67-positive CTC cohorts, but there was a significantly reduced OS in the vimentin-positive and Ki67-positive CTC cohorts compared to those patients with no vimentin/Ki67-positive CTCs, respectively. In both Ki67 and vimentin cohorts, no additional prognostic significance of Ki67- or vimentin-positive CTCs was evident when cohorts were subdivided into the unfavourable prognostic category of ≥5 standard CTCs overall, or when patients with high proportions of Ki67/vimentin-positive CTCs were compared to patients with high proportions of Ki67/vimentin-negative CTCs. This suggests that the presence of vimentin-positive or Ki67-positive CTC is more clinically important than their relative numbers or proportions, and that their presence in the early stages of advanced disease could be of greater prognostic significance than in later stages. Finally, there was no change in patient percentage or CTC proportion with Ki67- or vimentin-expressing CTCs before and after treatment, suggesting the potential of these techniques to offer predictive benefits of treatment remains unestablished.

It is important to reflect on the deficiencies of this dataset and its analysis. The results here are hypothesis-generating and should be interpreted within the context of a retrospective observational design. Survival data reported was not adjusted for patient treatment, and mCRPC patient numbers tested were relatively small, particularly for the Ki67 cohort. The variability of planned treatments was the reason progression-free survival was not evaluated in our study: with small patient numbers, an imbalance of treatment line and therapy was inevitable between our patient cohorts and would have confounded such an analysis. Finally, an initial biological selection during CellSearch should also be noted: it will only test for Ki67 and vimentin after patient CTC have been initially isolated using EPCAM-coated ferromagnetic beads, an epithelial-antibody based selection. This process is consistent across all patient cohorts analysed, but a fully representative picture of mesenchymal and proliferative CTCs may be impaired as a consequence. As an alternative to antibody-based CTC selection via CellSearch, a number of other CTC detection techniques are in different stages of development, some with the aim of isolating CTC by their large size rather than their biological phenotype: a similar analysis of CTC vimentin expression in mCRPC using these techniques would be of future interest, and could offer a more accurate picture of its clinical relevance.

We noted a significant reduction of OS in the patient cohort harbouring vimentin-expressing CTC: HR was 1.8, with a median of 305 days OS in the vimentin-positive CTC population compared to 453 days for those patients with no vimentin-positive CTCs. Given some of the study limitations noted in the previous paragraph (ie the possibility that a high percentage of mesenchymal CTC are missed by the CellSearch technology), this finding merits further examination and has future potential to offer important insight regarding mesenchymal CTC and their treatment. It is possible that this difference could have been more profound using CTC enrichment platforms that are not antibody-based. More opportunity to perform detailed study of vimentin co-localisation with other CTC markers should hopefully arise soon, given the recent identification and clinical characterisation of a non-commercial cell surface vimentin antibody that detects CTCs from epithelial cancers with high sensitivity and specificity [28].

Mesenchymal or EMT CTC, as represented by vimentin expression in this study, have previously been associated with tumour invasiveness, chemoresistance, CTC clustering, stem cell characteristics, and poorer clinical outcomes [29, 30]. Although not validated by our study, the results generated here are generally consistent with this proposed biological and clinical phenotype. In parallel with the poorer prognosis seen in this study for those patients with vimentin-positive CTCs at baseline, the lack of survival difference observed in those patients whose samples contained the highest proportion of vimentin-positive CTCs is consistent with (but does not confirm) the stem cell theory that just one vimentin-positive cell will suffice to contribute to poorer outcomes. Armstrong and colleagues previously used the CellSearch platform to offer a detailed biological examination of vimentin and other markers of the EMT program in samples from patients with breast and prostate cancer, revealing the existence and high frequency of CTCs that can co-express epithelial and mesenchymal proteins [25]. Using a cutoff of 1 vimentin-positive CTC, 30/93 CRPC patients from our study had baseline samples with CTCs that co-expressed cytokeratin with vimentin, compared to 10/10 CRPC patients documented previously. This disparity could be accounted for by differences between patient characteristics and staining methods employed in the two studies, but here we have also been able to build on the clinical aspect of vimentin analysis by offering the larger clinical cohort with longer-term follow-up that was recommended in the previous study.

A significant reduction of OS was also observed in the patient cohort harbouring Ki67-expressing CTC: HR was 2.37, with a median of 512 days OS in the Ki67-positive CTC population compared to 751 days for those patients with no Ki67-positive CTCs. One previous study has explored the biological character of Ki67-positive CTC using an alternative CTC isolation platform, also offering some preliminary insight from CTC enumeration of 10 patient samples: 12.3 % of CTCs were Ki67-positive in our study, compared to 7–73 % from 4 CRPC patients reported previously [18]. We have thus been able to consolidate the results of this initial study using larger patient numbers and the CellSearch platform, suggesting again that there is potential prognostic clinical insight to be gained by examining proliferative and non-proliferative CTC subpopulations. Future studies can now focus prospectively on prognostic validation of Ki67-positive CTCs and their response to particular treatment types.

A number of other prostate cancer CTC studies have recently elaborated on the original work which showed the prognostic value of CTC in mCRPC [4, 5]. In terms of CTC enumeration, a putative predictive role for ‘categorical’ CTC enumeration (ie whether a patient is CTC positive or not) has been suggested after 1 cycle of chemotherapy, an evaluation that can be performed weeks ahead of standard response approaches such as PSA and CT [31]. A recent large prospective study suggested that CTC count and LDH value could be a surrogate of overall survival in mCRPC treated with abiraterone [6]. On the other end of the research spectrum, the biological heterogeneity of prostate cancer with treatment has also recently been re-illustrated despite its seemingly predictable clinical pattern of metastatic spread compared to many other cancers: CTC measurement and next generation single cell sequencing of metastatic prostate cancer patient revealed a vast complexity of clonal evolution, a finding consistent with the metastasis-to-metastasis spread of multiple tumour clones recently revealed by prostate tumour biopsies [32–34]. Many of these studies have concentrated on androgen receptor expression and its predictive capacity [11, 12, 35]. Overall, the vast range of CTC enumeration, genomic and biological progress achieved in these studies offer an encouraging window of insight towards the future for circulating biomarkers in prostate cancer.

## Conclusions

Here we show that routine clinical testing of vimentin and Ki67 expression in CTC derived from mCRPC patients is feasible. A significant reduction in OS for patients with baseline CTC vimentin and Ki67 expression suggests that largescale prospective analyses would be important to further explore these potential links. Future studies will also require further detailed correlation of vimentin/Ki67 CTC expression and response to certain treatment types: for example, the predictive capacity of lost or increased Ki67 CTC-expression in a large cohort of mCRPC patients treated exclusively with anti-proliferative chemotherapy agents such as docetaxel or cabazitaxel. Finally, a number of alternative CTC-isolation techniques have been developed and merit further analysis for such clinical correlation studies: size-based filtration techniques may be particularly helpful for vimentin-expressing CTC which may not be efficiently captured by techniques based on epithelial-antibody expression.

## Additional file

Additional file 1: Figure S1. Validation of vimentin and Ki67 staining using the CellSearch platform. **Figure S2.** Prognostic impact of total CTC in two cohorts of patients with CRPC. **Figure S3.** Prognostic impact of vimentin/Ki67 CTC proportions and their presence in the unfavourable subgroup. Important validation work performed to justify subsequent experiments and conclusions. **Figure S1.** details the optimisation on CellSearch of Vimentin and Ki67 antibodies. **Figure S2.** shows that prognostic analysis of total CTC (regardless of vimentin and Ki67 measurements) is consistent with previous reports.** Figure S3.** shows that there is no prognostic significance of KI67 or vimentin CTC in the unfavourable prognosis subgroup, and that proportion of Ki67- or vimentin-CTC is less prognostically important than their presence or absence. (PDF 1030 kb)

## Abbreviations

AR: androgen receptor
CRPCcastrate-resistant: prostate cancer
CTCcirculating: tumor cell
EMTepithelial-mesenchymal: transition
HR: hazard ratio
IF: immuno-fluorescence
mCRPC: metastatic castrate-resistant prostate cancer
OS: overall survival
PSA: prostate-specific antigen
RTOG: Radiation Therapy Oncology Group
