# Supplementary material for: Vimentin and Ki67 expression in circulating tumour cells derived from castrate-resistant prostate cancer
Source: BMC Cancer. 2016 Feb 29;16:168. doi: 10.1186/s12885-016-2192-6 (PMC4770547; doi:10.1186/s12885-016-2192-6)
Supplement: Additional file 1: Figure S1. — Validation of vimentin and Ki67 staining using the CellSearch platform. Figure S2. Prognostic impact of total CTC in two cohorts of patients with CRPC. Figure S3. Prognostic impact of vimentin/Ki67 CTC proportions and their presence in the unfavourable subgroup. Important validation work performed to justify subsequent experiments and conclusions. Figure S1. details the optimisation on CellSearch of Vimentin and Ki67 antibodies. Figure S2. shows that prognostic analysis of total CTC (regardless of vimentin and Ki67 measurements) is consistent with previous reports. Figure S3. shows that there is no prognostic significance of KI67 or vimentin CTC in the unfavourable prognosis subgroup, and that proportion of Ki67- or vimentin-CTC is less prognostically important than their presence or absence. (PDF 1030 kb) [file 12885_2016_2192_MOESM1_ESM.pdf]

Vimentin and Ki67 expression in circulating tumour cells derived from castrate-resistant prostate cancer

Additional Files

C.R. Lindsay[1]*, S. Le Moulec[2]*, F. Billiot[1], Y. Loriot[3], M. Ngo-Camus[3], Vielh P[1,4], K. Fizazi[3], C. Massard[3]*, F. Farace[1]*#

*these authors contributed equally to the publication

# corresponding author

1. INSERM U981, University of Paris-Sud XI, Translational Research Laboratory, Gustave Roussy Cancer Campus, Villejuif, France.
2. Department of Medical Oncology, Val de Grâce Hospital, Paris, France.
3. Department of Medical Oncology, Gustave Roussy Cancer Campus, Villejuif, France.

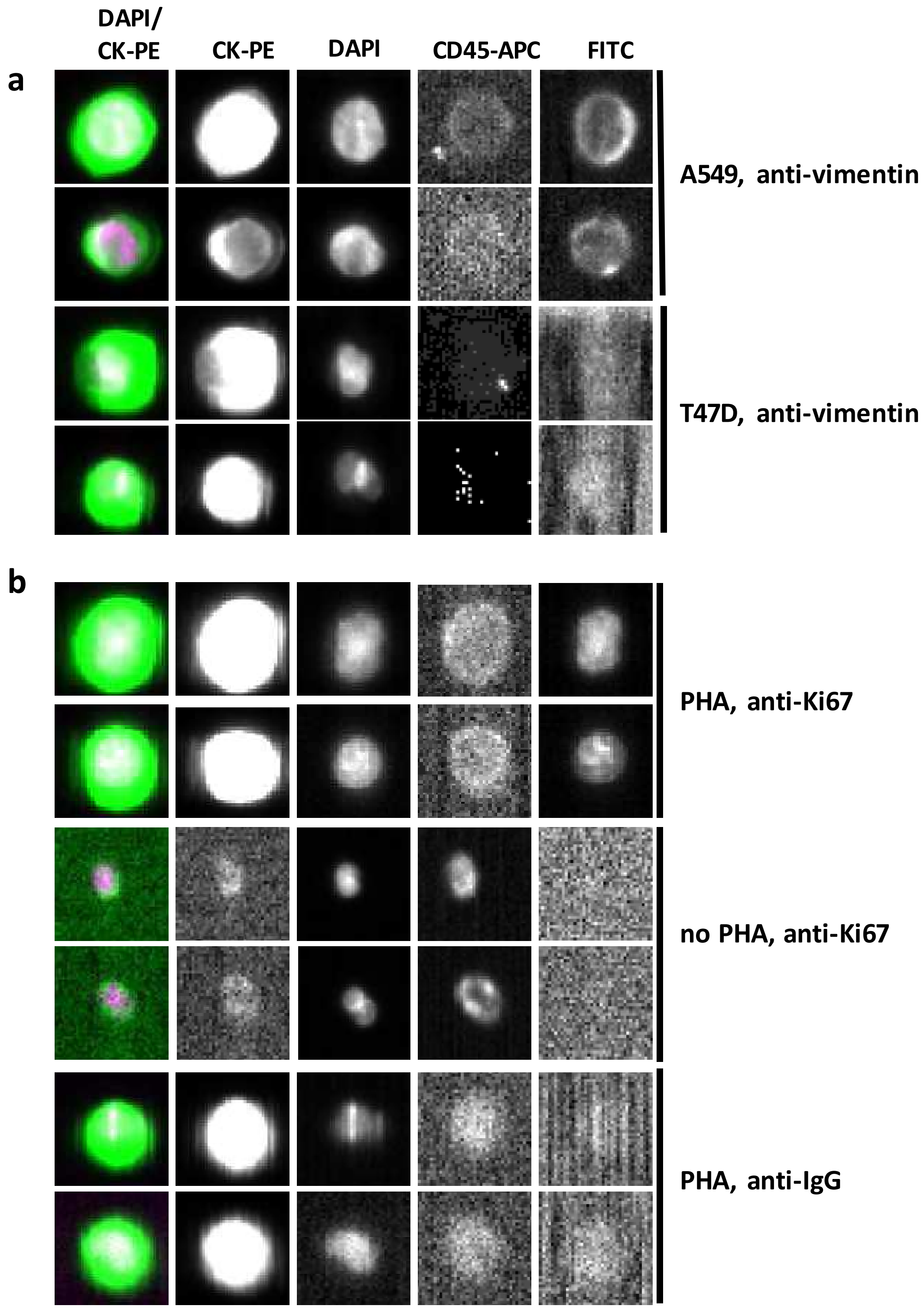

Supplementary Figure 1 Validation of vimentin and Ki67 staining using the CellSearch platform a) Representative pictures from CellSearch system analyses of vimentin staining in A549 cells (vimentin +ve) and T47D cells (vimentin - ve) using the free channel (FITC). b) Representative pictures from CellSearch system analyses of Ki67 staining in A549 cells using the free channel (FITC). PHA was added to cells in the top and bottom panels to stimulate proliferation. PHA = phytohaemagglutinin A.

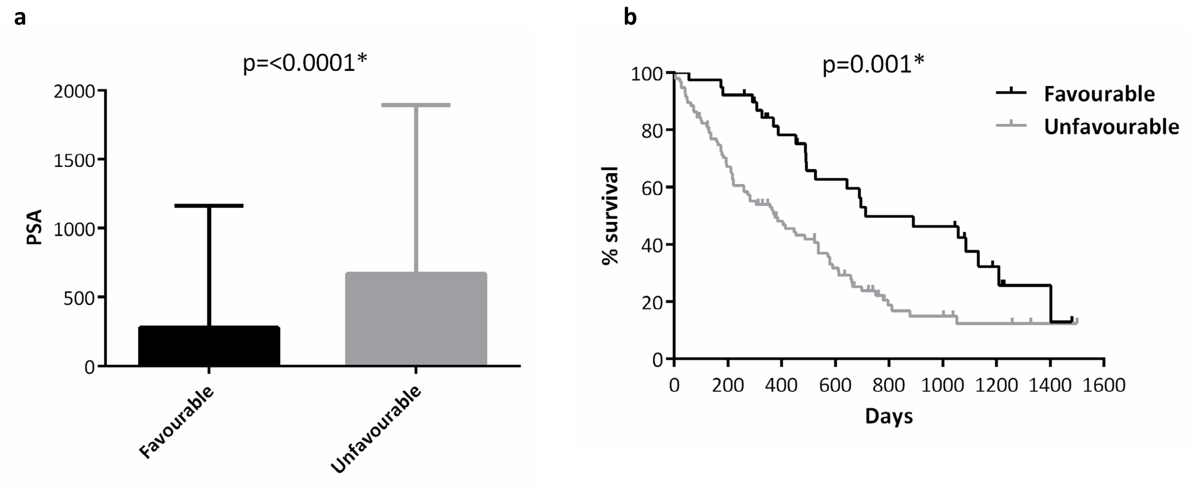

Supplementary Figure 2 Prognostic impact of standard CTCs in two cohorts of patients with CRPC Prognostic comparison of unfavourable category of patients at baseline (≥5 standard CTCs overall) compared to favourable category (<5 standard CTCs overall), as assessed by bar charts comparing baseline PSA (a) and Kaplan-Meier plots of overall survival (b). Mann-Whitney test performed for (a) and log-rank tests for (b); Bar chart shows mean +/- standard deviation, p=<0.05.

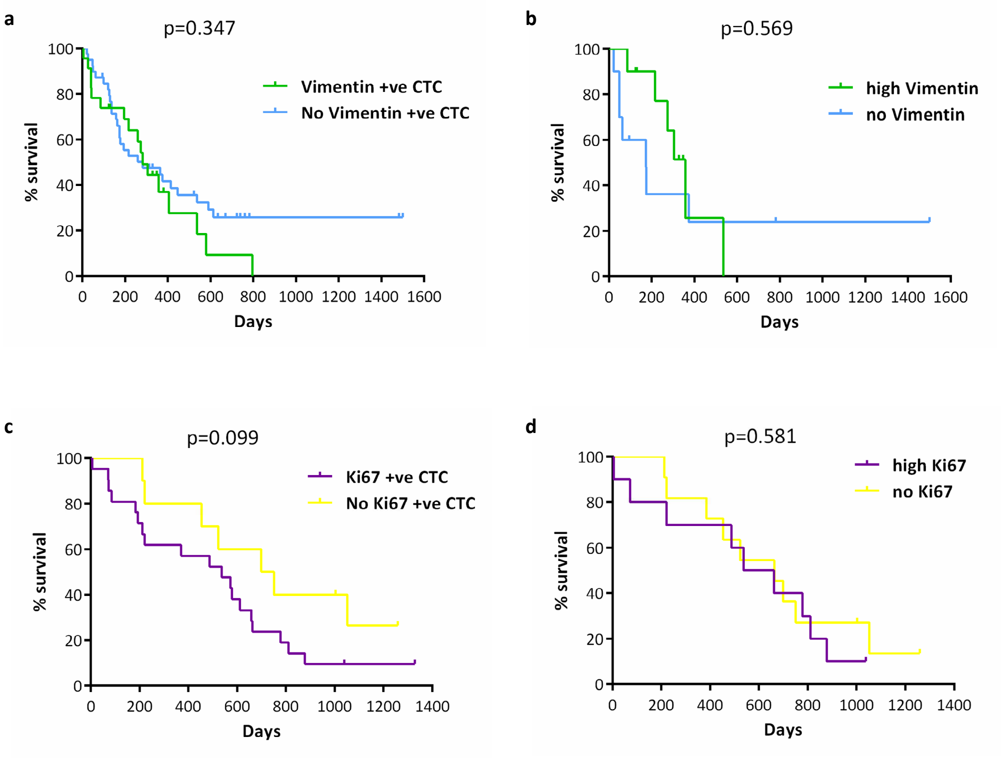

Supplementary Figure 3 Prognostic impact of vimentin/Ki67 CTC proportions and their presence in the unfavourable subgroup (a) Kaplan-Meier survival of patients with ≥1 vimentin-positive CTC in unfavourable subgroup (≥5 standard CTCs overall) at baseline compared to those in this subgroup with no vimentin-positive CTC (b) Kaplan-Meier survival of patients with highest proportion of vimentin-positive CTC at baseline compared to those with the highest proportion of vimentin-negative CTC (c) Kaplan-Meier survival of patients with ≥1 Ki67-positive CTC in unfavourable subgroup (≥5 standard CTCs overall) at baseline compared to those with no Ki67-positive CTC (d) Kaplan-Meier survival of patients with highest proportion of Ki67-positive CTC at baseline compared to those with the highest proportion of Ki67-negative CTC. Log-rank tests for all; p=<0.05.
